# Correlates of COVID-19 Preventative Behaviors before and after Vaccination Availability

**DOI:** 10.3390/bs13060501

**Published:** 2023-06-14

**Authors:** Kristopher J. Kimbler, Caleb Gromer, Melissa Ayala, Brianna Casey

**Affiliations:** Psychology Department, Florida Gulf Coast University, Fort Myers, FL 33965, USA

**Keywords:** COVID-19, preventative health behaviors, interpersonal concern, normative beliefs, personality, political beliefs, public health

## Abstract

As the COVID-19 pandemic progressed, various preventative behaviors and eventually vaccinations became available to decrease the spread of the virus. The current study examined a variety of variables (i.e., age, COVID-19-related economic hardship, interpersonal concern, personality, fear of COVID-19, normative beliefs, political beliefs, and vaccine hesitancy) to better understand predictors of preventative behaviors and vaccination status at different points throughout the pandemic. Online questionnaires, administered through Qualtrics, were used to collect data using two convenience samples. One was a small sample (*N* = 44) of non-student participants before the vaccine was readily available. The other sample (*N* = 274) included college student participants and occurred after the vaccine had been available to all participants. Results suggest that several variables (i.e., fear of COVID-19, normative beliefs, interpersonal concern, and openness) were consistent predictors of public health behaviors at both points in time and across differently aged samples. Other variables (i.e., agreeableness, extraversion, conscientiousness, and economic hardship) were less consistent with their relationships with public health behaviors. Implications related to both research and public health are discussed.

## 1. Introduction

Coronavirus Disease-2019 (COVID-19) quickly spread throughout the world, causing a large number of deaths and serious illnesses. To slow the spread, to prevent further deaths, and to keep from overwhelming the hospital systems, an emphasis was placed on preventative behaviors [1]. Practicing preventive behaviors such as mask-wearing, avoiding large crowds, hand washing, and social distancing became essential to slowing the spread of the virus. A major obstacle to preventing the spread of COVID-19 was a lack of compliance by many [2], resulting in a greater need to better understand predictors of whether individuals are willing to practice preventative behaviors. Once COVID-19 vaccines were disseminated, vaccine hesitancy became an additional threat to public health efforts [3]. It is therefore important to better understand the various potential predictors of compliance for preventative behaviors, including vaccinations. The current study focuses on better understanding the role of age, interpersonal concern, COVID-19-related economic hardship, fear of COVID-19, normative beliefs, pre-existing conditions, personality, vaccine hesitancy, and political beliefs to help further understand the various potential influences in predicting behavioral components of public health.

### 1.1. Age

Previous findings consistently demonstrate that the risk of serious illness and death is closely associated with age, as older adults have a much higher likelihood of becoming seriously ill and/or dying after contracting COVID-19 [4]. This increased risk would logically suggest that older adults would demonstrate a higher prevalence of preventative behaviors. According to Kim and Crimmins [5], older individuals (over age 55) initially did not differ from younger individuals related to preventative behaviors, but a month into the pandemic, older adults were significantly more likely to engage in preventative behaviors. Similarly, older adults exhibited less high-risk behaviors, though both younger and older adults engaged in more high-risk behaviors as the pandemic progressed. Research has also demonstrated that younger males were associated with lower levels of preventive behaviors compared to other groups [6]. While research examining the relationship between age and preventative behaviors tends to find that, due to the increased risk, older adults are more likely to exhibit preventative behaviors, findings have not always been consistent. For example, Clark and colleagues [2] found that age was not related to following COVID-19 rule compliance and was only weakly associated with preventative behaviors.

### 1.2. Interpersonal Concern

Intrapersonal concern involves the extent that individuals take into consideration how their behaviors may affect those around them or society overall. Interpersonal concern has been found to predict preventative behaviors related to other infectious diseases such as tuberculosis [7]. While previous research has not explicitly studied the relation between interpersonal concern and COVID-19, other research suggests a possible relationship. Cultural influences are likely associated with interpersonal concern. Within individualistic cultures, individuals are taught to value the individual over the group, whereas in collectivist cultures, individuals are taught to value the group over the individual [8]. Previous research [9,10] has found that collectivistic countries exhibited greater levels of COVID-19 preventative behaviors. Additionally, variance in collectivism within countries was associated with increased levels of preventative COVID-19 behaviors. This research has demonstrated that variables indicating a concern for others tend to be more predictive of preventative behaviors than variables indicating more self-focus [11]. This variation in the extent that individuals prioritize the self or others could also indicate different strategies when promoting preventative behaviors and public health. For example, research conducted in individualistic societies has demonstrated that participants were more likely to respond positively to health messages about social distancing when they were framed as benefiting the individual rather than others [12].

It is important to note, however, that individualistic and collectivistic cultures may differ related to variables other than general concern for others. While research specifically examining the role of interpersonal concerns and COVID-19 preventative behaviors is lacking, some research suggests that concern for others may be important in understanding preventative behaviors related to COVID-19. Qualitative research [13] demonstrates that individuals indicated a concern for others as a reason for practicing COVID-19 preventative behaviors when those behaviors were uncomfortable or inconvenient to the individual. 

### 1.3. Economic Hardship 

COVID-19 is not only a virus that has affected global health, but it also had an adverse effect on the economy. Another COVID-19 issue to consider is whether economic factors are also related to preventative behaviors. One study [14] found that income was not directly associated with preventive behaviors. Other research has indicated that those in higher income brackets are more likely to exhibit preventative behaviors than those earning lower amounts [6,15,16]. While these findings are mixed, research typically has suggested that lower incomes are associated with less preventative behaviors.

While this research suggests that lower income is associated with a decreased likelihood of practicing preventative behaviors, there could be a variety of potential mechanisms driving this relationship. Some research suggests that participants with higher incomes were more likely to stay home during the pandemic compared to participants with lower incomes [17]. One reason that income may be predictive of staying home is the types of occupations more common in lower- and upper-income households. Research using mobility tracking in high- and low-income neighborhoods suggests that both groups left the house a similar amount for the purposes of shopping, recreation, and healthcare. Low-income neighborhoods, however, were more likely to leave home for work, while individuals in high-income neighborhoods were more likely to work from home, suggesting that occupational differences could explain some of the differences in income-based risk.

While differences based on income are important, another potentially important variable that has been addressed less in the research is the negative influence that COVID-19 played on the economy. Since there were several negative economic influences related to COVID-19, it is important to understand whether experiencing economic hardship as a result of the pandemic relates to changes in preventative behaviors. Lemay and colleagues [18] examined the role of health and economic threats in relation to COVID-19. Participants experiencing health threats related to COVID-19 were more likely to respond with increased communal values, leading to preventative behaviors. Participants experiencing economic threats (as indicated by a collection of economic variables, including the extent that participants perceived economic hardship as a result of COVID-19) focused more on individual control and achievement, leading to less preventative behaviors. It therefore seems plausible that the relationship between income and economic hardship is due to a variety of factors such as perceived threats and occupational requirements.

### 1.4. Fear of COVID-19 

Due to the potential for serious negative health outcomes caused by COVID-19, fear of COVID-19 is another potentially motivating factor related to a variety of issues involving preventative behaviors. For example, participants reporting pre-existing health conditions or poor health have also reported greater fear of COVID-19, presumably due to their increased risk of serious illness [19]. Other research has consistently demonstrated a positive relationship between fear of COVID-19 and increased preventative behaviors [20,21,22].

### 1.5. Normative Beliefs 

Normative beliefs, or the extent that individuals perceive specific behaviors as accepted within their social circle or society, have been found to predict a variety of health behaviors. For example, perceptions of smoking approval among family and friends are predictive of smoking behavior after accounting for a variety of other variables [23]. Regarding COVID-19, perceived norms have been found to predict preventative behaviors [24]. Research has demonstrated that perceptions of beliefs held by family and peers were more predictive of preventative behaviors than overall societal norms [25]. Experimental research related to COVID-19 has demonstrated that preventative health decisions were affected by both normative behavior (the extent that peers are attending a large gathering) and normative beliefs (the extent that peers approve of attending large gatherings). When participants were exposed to either high levels of gathering attendance or normative approval of attending large events, they were less likely to report avoiding the large crowd [26]. Correlational research examining perceived norms related to COVID-19 and preventative behaviors is lacking.

### 1.6. Pre-Existing Conditions

Research has consistently demonstrated that people with certain pre-existing conditions have a higher risk of serious illness and death associated with COVID-19 compared to people without these pre-existing conditions [27]. Research has also consistently demonstrated that having pre-existing conditions result in significantly increased fear of COVID-19 [19,28]. Considering these individuals are at greater risk of serious illness, are more fearful of COVID-19, and (as described earlier) fear of COVID-19 is associated with more preventative behavior, it seems plausible that having one of these pre-existing conditions would be a motivating factor in preventative behaviors. Surprisingly, however, findings are mixed. For example, some studies have found that increased risk due to pre-existing conditions does not consistently predict preventative behaviors [28]. Porteny and colleagues [29] found a more nuanced relationship between pre-existing conditions and preventative behaviors. Specifically, only individuals with obesity combined with other high-risk pre-existing conditions were found to exhibit increased preventative behaviors. Participants reporting only obesity or pre-existing conditions that did not include obesity were no more likely to exhibit preventative behaviors than those without any pre-existing conditions. 

### 1.7. Personality

Examining the relation between personality and preventative health behaviors has demonstrated several significant findings. For example, individuals high in agreeableness tend to exhibit more preventative behaviors related to contagious diseases [7]. Specific to COVID-19, research has consistently demonstrated that higher levels of agreeableness and conscientiousness are related to increased preventative health behaviors [30,31]. Other personality characteristics have been less consistently associated with COVID-19 preventative behaviors. For example, neuroticism and openness have been linked to practicing social distancing behaviors, whereas extraversion was negatively associated with following social distancing protocols [30]. While Ludeke and colleagues found more significant relationships between personality and preventative behaviors, it is important to point out that this study had a very large sample (*N* = 89,305) and the effect sizes related to personality were small. Yet, other research has demonstrated contradicting findings. While Al-Omiri and others [32] replicated other studies related to agreeableness and conscientiousness, they found that high levels of extroversion were associated with more acceptance of efforts to contain the spread of COVID-19. Similarly, other studies have identified positive relationships between extraversion and preventative behaviors such as social distancing [33]. Other research has failed to find any relationship between extraversion and preventative behaviors [34]. Findings related to personality and COVID-19 preventative behaviors have therefore exhibited somewhat consistent findings related to some aspects of personality (e.g., agreeableness and conscientiousness), but mixed findings related to extraversion.

### 1.8. Political Beliefs 

Research indicates that efforts at promoting public health have been largely politicized during the COVID-19 pandemic. For example, participants’ political voting preference (i.e., voting for Donald Trump) has been demonstrated to predict fewer preventive behaviors than the participants who voted for Joe Biden [29]. Other research examining differences in COVID-19 behaviors and outcomes suggests that participants with right leaning political views were more likely to test positive for COVID-19, more likely to report believing in COVID-19 conspiracy theories, and less likely to practice preventative behaviors. These findings support the idea that conservatives are less likely to practice preventive behaviors than liberals and that those behavioral differences likely result in higher rates of infection. Experimental research has provided additional support for how politics relate to public health during the COVID-19 pandemic. One such study [35] examined the effects of a pro-social distancing message. The results showed that when the message was delivered by a Republican, the trust among conservatives increased. 

### 1.9. Specific Aims

To better understand how the previously described variables relate to preventative behaviors and vaccination status, the current study addressed several specific aims. Study 1 assessed whether interpersonal concern, economic hardship, fear of COVID-19, normative beliefs, pre-existing conditions, and personality were associated with preventative behaviors and vaccination plans before vaccines were available. Study 2 assessed whether interpersonal concern, economic hardship, fear of COVID-19, normative beliefs, pre-existing conditions, personality, and vaccine hesitancy were associated with preventative behaviors and vaccination status after vaccines were available and whether these relationships differed when examining peak and current preventative behaviors. 

## 2. Materials and Methods

The current study includes two separate data collections, which differ somewhat in methodology and outcomes due to the changing nature of the COVID-19 pandemic. Data for Study 1 were collected from August 2020 until March 2021. This covered a time period before vaccinations were approved as well as a time period after approval when vaccines were not readily available. Study 2 took place from August 2021 until November of 2021. At this point, all adults were eligible for vaccinations and vaccines were free and readily available. Ethical approval for this research was provided by the Internal Review Board (IRB) at Florida Gulf Coast University.

### 2.1. Sample Characteristics

#### 2.1.1. Study 1

A convenience sample of participants was collected using participant referrals and snowballing techniques to recruit a non-student sample of participants (*N* = 44). Participants were diverse related to age, ranging from age 18 to 87 (*M* = 38.70; *SD* = 21.95). More participants identified as women (63.64%) compared to men (31.82%). Regarding race/ethnicity, 75.00% of the sample indicated that they are white/Caucasian, 9.09% identified as Latinx, 4.55% identified as black/African American, and 2.27% reported other. The political affiliation of this sample was 54.55% Republican, 20.45% Democrat, and 20.45% identified as having ‘other’ political affiliation.

#### 2.1.2. Study 2

In attempt to obtain a larger sample size, in addition to the referrals, undergraduate psychology students were also recruited. This resulted in a larger sample size (*N* = 274). Participants age range was 18–61 (*M* = 19.99; *SD* = 6.30). The sample was predominately women (81.0% women; 17.6% men; 1.5% Other). Regarding race/ethnicity, 68.5% reported white/Caucasian, 19.8% reported Latinx, 10.3% reported black/African American, 1.1% reported Asian, and 0.4% reported other. The sample for Study 2 consisted of more Democrats (34.2%), with 29.8% identifying as Republicans, and 35.0% reporting other political affiliations.

### 2.2. Procedures

While there were deviations in the procedures for Study 1 and Study 2, the majority of procedures and measures were consistent. Participants completed an online questionnaire administered through Qualtrics. Participants first completed informed consent. They then completed measures related to interpersonal concern, COVID-19-related economic hardship, fear of COVID-19, personality, experiences with COVID-19, participant characteristics, normative beliefs, preventative behaviors, and pre-existing conditions (all measures described below). These components of the study were similar throughout both samples.

#### 2.2.1. Unique Aspects of Study 1

These data were collected before any vaccines were approved or readily available. In addition to completing the list of measures above, participants then indicated their vaccination plans. 

#### 2.2.2. Unique Aspects of Study 2

There was a 4-month break in data collection between Study 1 and Study 2. During this time vaccines became readily available for all adults. For Study 2, the vaccination aspect of the preventative behaviors focused on vaccination status (instead of plans) since anyone that wanted a vaccine could have received one by this time. Participants simply indicated whether or not they had been vaccinated for COVID-19. Additionally, due to the availability of vaccines, many COVID-19 restrictions had been lifted, masks were no longer required in many public places, and university campuses resumed to normal activities with typical class sizes as no mandates were in place regarding preventative behaviors. As a result, people were practicing fewer preventative behaviors compared to the prior months. Due to this, another change in the procedures related to preventative behaviors was made. In addition to reporting their current levels of preventative behaviors (similar to Study 1), participants also reported the extent that they exhibited preventative behaviors during the time period when they were being the most cautious related to COVID-19. Finally, since vaccines were readily available during Study 2, an additional area of interest involved vaccine hesitancy. 

### 2.3. Measures

#### 2.3.1. Demographics

After completing the informed consent, participants completed a brief demographics questionnaire. They were asked to provide information regarding their age, gender, race/ethnicity, economic status, employment status, and political affiliation.

#### 2.3.2. Preventative Behaviors/Vaccines

Participants also provided information related to preventative behaviors. They rated, on a 5-point Likert scale, 4 questions asking about the extent that they wear a mask in public, avoid large crowds, wash hands when in public places, and maintain social distancing. For Study 2, these 4 questions were also asked related to when participants were practicing their peak level of precautions related to COVID-19. In addition to these preventative behaviors, participants in Study 1 also rated (using a 5-point scale) their likelihood of getting a vaccine. This was performed using a 5-point Likert scale ranging from ‘definitely not planning on getting the vaccine’ to ‘definitely planning on getting the vaccine’. For Study 2, after vaccines were readily available, participants indicated (yes or no) whether or not they had been vaccinated.

#### 2.3.3. Interpersonal Concern 

The extent that individuals are concerned about others or society at large was assessed using a 6-item scale previously used in research [7]. This measure asked participants the extent that they agree with 6 statements (using a 5-point Likert scale) that indicate concern for others (e.g., ‘We should care for each other more in public places).

#### 2.3.4. COVID-19-Related Economic Hardship

To better understand the extent that participants perceived economic harm as a result of COVID-19, they were asked to rate (on a 5-point Likert scale) the extent that they suffered economic hardship that they attributed to COVID-19. The 6 items used to assess this economic hardship included the extent that they lost a job, had work hours decreased, had a decrease in pay, experienced a decrease in general savings, and experienced a decrease in retirement savings that they attributed to COVID-19.

#### 2.3.5. Fear of COVID-19

To determine the extent that participants were fearful of COVID-19, the Fear of COVID-19 scale was administered [36]. This measure consists of 7 items describing fearful responses to COVID-19 (e.g., I am afraid of dying because of coronavirus-19). Participants rated the extent that they agree with each statement using a 5-point Likert scale.

#### 2.3.6. Personality

The Big 5 Inventory [37] was used to measure personality. For this questionnaire, participants rate (on a 5-point Likert scale) the extent that they agree with 44 items that indicate different aspect of the big 5 personality traits. This results in a score for extraversion, agreeableness, conscientiousness, neuroticism, and openness.

#### 2.3.7. Experiences with COVID-19

Participants were asked a series of yes or no questions related to their experience with COVID-19. Participants were considered to have mild experiences with COVID-19 if they indicated that they had been tested, had tested positive (but without hospitalization), or knew someone personally that tested positive (without hospitalization). Participants were considered to have severe experience with COVID-19 if they indicated they had been hospitalized, knew someone personally that had been hospitalized, or knew someone personally that had died from COVID-19.

#### 2.3.8. Normative Beliefs

To assess participants’ normative beliefs regarding COVID-19 preventative behaviors, a 12-item questionnaire was developed. This measure closely modeled a questionnaire used in previous research to assess normative beliefs related to smoking behaviors [23] by changing the smoking behaviors to COVID-19 preventative behaviors. Each item asked about the extent (scored on a 5-point Likert scale) that individuals from 1 of 3 groups of people (friends, family, and ‘people I admire or look up to’) exhibit 4 different COVID-19 preventative behaviors. The 4 preventative behaviors (wearing a mask in public, avoiding large crowds, washing hands when in public places, and maintaining social distancing) were the same 4 preventative behaviors that were previously reported for individual participant preventative behaviors. 

#### 2.3.9. Pre-Existing Conditions

Participants were asked how many pre-existing conditions they have that could place them at a higher risk of contracting COVID-19. They were provided with a list of conditions that the CDC had identified as pre-existing conditions that place individuals at a higher risk of experiencing serious illness from COVID-19. These conditions included cancer, chronic kidney disease, COPD (chronic obstructive pulmonary disease), heart conditions, an immunocompromised state (weakened immune system), obesity, severe obesity, sickle cell disease, smoking, and type-2 diabetes. They were also presented with a list of conditions that the CDC had indicated could potentially increase the risk of serious illness. This list included asthma, cerebrovascular disease, cystic fibrosis, hypertension (high blood pressure), neurologic conditions, liver disease, overweight, pregnancy, pulmonary fibrosis, thalassemia, and type-1 diabetes. For each of the 2 lists, participants indicated whether they had none, 1, 2, 3, or 4 or more of these conditions to obtain a general indication about the extent that individuals were at an increased risk of COVID-19 without asking about specific health conditions.

#### 2.3.10. Vaccine Hesitancy

Considering vaccines were readily available for Study 2, an additional measure was included to assess reasons for vaccine hesitancy. This measure was developed based on a vaccine hesitancy questionnaire previously used to measure the extent that individuals were hesitant to get vaccinated for HPV [38]. Some of the items from the original measure were generalizable to the COVID-19 vaccine (e.g., not liking shots, fear of side effects). Other items were removed as they related specifically to the HPV vaccine such as items about promoting sexual promiscuity. Additionally, new COVID-19 specific items were included that are specific to both concerns and conspiracy theories that individuals reported as impediments to vaccinations. The content of these items originated from the CDC addressing frequently asked questions about the vaccine [39]. The COVID-19 specific items that were added to the original measure included: COVID-19 not being severe enough to warrant vaccination, the vaccine being more dangerous than COVID-19, the vaccine implants microchips, and the vaccine makes people magnetic. This resulted in a 21-item questionnaire that listed several statements about reasons someone would not want to get vaccinated. Participants rated their agreement with each item using a 5-point Likert scale. The vaccine hesitancy questionnaire was administered to all participants during Study 2, regardless of their vaccination status since some vaccinated individuals may have also had reservations about getting the vaccination.

## 3. Results

### 3.1. Study 1 (N = 44)

To test the extent that vaccine plans and preventative behaviors were predicted by interpersonal concern, COVID-19-related economic hardship, fear of COVID-19, normative beliefs, pre-existing conditions, and personality, bivariate Pearson’s correlations were conducted (See Table 1). Generally, these variables were more predictive of preventative behaviors than vaccine plans. Individuals were more likely to exhibit a high amount of preventative behaviors if they planned to get vaccinated (*r* = 0.35; *p* < 0.05), were older (*r* = 0.53; *p* < 0.01), had high levels of interpersonal concern (*r* = 0.34; *p* < 0.05), experienced less COVID-19-related economic hardship (*r* = −0.40; *p* < 0.05), were more fearful of COVID-19 (*r* = 0.58; *p* < 0.01), had beliefs that preventative behaviors were normative (*r* = 0.79; *p* < 0.01), and were open to new experiences (*r* = 0.51; *p* < 0.01). Planning to get vaccinated was predicted by fear of COVID-19 (*r* = 0.42; *p* < 0.01), having beliefs that preventative behaviors are normative (*r* = 0.37; *p* < 0.05), and being agreeable (*r* = 0.44; *p* < 0.01).

Participants were also categorized into three groups based on their experiences with COVID-19. One group included participants with severe experiences related to COVID-19 (having been hospitalized, knowing someone that was hospitalized, or knowing someone that has died). The second group consisted of individuals with mild experiences related to COVID-19 (having been tested, testing positive but without hospitalization, or knowing someone who tested positive without hospitalization). The third group was participants with no experience with COVID-19 (knowing no one that has tested positive and having never been tested for COVID-19). Two Analysis of Variance (ANOVA) tests were conducted to examine group differences based on experience with COVID-19 in preventative behaviors and vaccination plans. There was a significant main effect of experiences with COVID-19, *F*(2,38) = 5.43, *p* = 0.01. Follow-up analyses revealed that participants with severe experiences related to COVID-19 reported fewer preventative behaviors (*M* = 2.27) compared to those with mild (*M* = 4.48; *p* < 0.05) or no (*M* = 4.01; *p* > 0.05) experience with COVID-19. Experiences with COVID-19 were not significantly related to vaccination plans (*p* > 0.05).

### 3.2. Study 2 (N = 274)

Similar to Study 1, bivariate correlations were conducted to determine whether the variables of interest relate to both preventative behaviors and peak preventative behaviors (see Table 2). Among the predominately college-aged sample, with a mean age of 19.99, age was negatively related to reports of peak preventative behaviors (*r* = −0.21; *p* < 0.05) but was not significantly related to current preventative behaviors. Interpersonal concern was positively correlated with both peak (*r* = 0.43; *p* < 0.01) and current preventative behaviors (*r* = 0.41; *p* < 0.01). Fear of COVID-19, normative beliefs, agreeableness, and openness were all also positively correlated with both peak and current preventative behaviors (*p* < 0.01). Conscientiousness was positively related to reports of both peak and current preventative behaviors, however, only the relationship with peak preventative behaviors was significant (*r* = 0.17; *p* < 0.05). Extraversion was not strongly associated with peak preventative behaviors (*r* = 0.03; *p* > 0.05) but was negatively related to current preventative behaviors (*r* = −0.21; *p* < 0.05). Vaccine hesitancy was negatively related to both peak (*r* = −0.27; *p* < 0.01) and current preventative behaviors (*r* = −0.30; *p* < 0.01).

To better understand the extent that this group of variables predict both peak and current preventative behaviors, linear regression analyses were conducted (See Table 3). The regression model included age, economic hardship, pre-existing conditions, interpersonal concern, personality, fear of COVID-19, normative beliefs, and vaccine hesitancy. The full model accounted for 57% of the variance in peak preventative behaviors. Interpersonal concern, conscientiousness, openness, normative beliefs, and vaccine hesitancy were all significant individual predictors (*p* < 0.05). The full model accounted for 66% of the variance in current preventative behaviors. Extraversion, conscientiousness, openness, fear of COVID-19, normative beliefs, and vaccine hesitancy were all significant individual predictors.

A series of *t*-tests were also conducted to determine whether vaccinated and unvaccinated individuals differed in any variables included in the regression above. Results indicated that vaccinated individuals scored significantly higher on fear of COVID-19, normative beliefs, and preventative behaviors (current and peak), but lower on conscientiousness, openness, and vaccine hesitancy (*p <* 0.05; See Table 4). Political affiliation also related to vaccination status (*χ*^2^ = 31.08; *p* < 0.01). Standardized residuals indicated that Democrats were more likely to be vaccinated while Republicans were less likely.

## 4. Discussion

This study identified several variables related to both vaccination status and preventative behaviors, as most relationships were in the anticipated direction. Data collected prior to the availability of vaccinations from a non-college student sample indicated that the likelihood of practicing preventative behaviors were predicted by being older and having higher levels of interpersonal concern, fear of COVID-19, normative beliefs, and openness. Preventative behaviors were negatively associated with COVID-19-related economic hardship. Vaccine plans were positively related to fear of COVID-19, normative beliefs, and agreeableness.

Data collected once vaccines were readily available (in a mostly college-student sample) indicated that both current and peak levels of preventative behaviors were positively related to interpersonal concern, fear of COVID-19, normative beliefs, agreeableness, and openness. Both current and peak preventative behaviors were negatively correlated with vaccine hesitancy. In addition to these consistent findings, peak preventative behaviors were also positively related to conscientiousness and negatively associated with age. Current preventative behaviors were also negatively correlated with extraversion.

Regression analyses revealed that when including all variables in the model, interpersonal concern, conscientiousness, openness, normative beliefs, and vaccine hesitancy were significant predictors of peak preventative behaviors. In the model predicting current preventative behaviors, extraversion, conscientiousness, openness, fear of COVID-19, normative beliefs, and vaccine hesitancy were all significant predictors. Additionally, vaccinated individuals were more likely to be Democrats and also reported more fear of COVID-19, more normative beliefs about preventative behaviors, and more preventative behaviors. Vaccinated individuals scored lower on conscientiousness, openness, and vaccine hesitancy.

The current study contributes to the understanding of variables related to whether individuals will engage in public health practices such as practicing preventative behaviors and becoming vaccinated for COVID-19. Findings from diversely-aged samples (Study 1) indicate that being older was associated with more preventative behaviors. This has been demonstrated in previous research [5] and would be expected given the tendency for older adults to be more likely to suffer serious health outcomes related to COVID-19 [4]. While increased age was associated with more preventative behaviors, age was not related to vaccination plans in the earlier days of the pandemic before vaccines were available. In the mostly college-aged sample, age was negatively correlated with preventative behaviors, however, this sample was young, with a mean age less than 20. It is possible that older participants within this sample were more likely to live away from their parents, having more freedom to ignore preventative behavior guidelines.

Interpersonal concern had been less explicitly addressed in the literature. Previous cross-cultural research has revealed that countries higher in collectivism tend to practice more preventative behaviors [10] compared to individualistic countries. The current study extends these findings by demonstrating that higher levels of within-culture interpersonal concern was also associated with more preventative behaviors. While interpersonal concern predicted preventative behaviors such as social distancing or wearing a mask, it was not associated with vaccination plans or vaccination status. This suggests that efforts to increase vaccinations should focus on how getting vaccinated could be beneficial to others in addition to preventing the individual from illness.

Financial status is also an interesting factor associated with preventative behaviors and vaccination status. Most research has focused on general economic conditions such as annual income [15] being positively associated with preventative behaviors. This study, however, focused specifically on the extent that individuals perceived economic difficulty as a result of the changes associated with COVID-19, which has been less addressed within the literature. Findings indicated that in the earlier stages of the pandemic, before vaccines were available, experiencing economic hardship as a result of COVID-19 was negatively related to both preventative behaviors and vaccination plans. There are several potential interpretations of these data. One possibility is that individuals that do not practice preventative behaviors may have been more likely to miss work due to contracting or being exposed to COVID-19, resulting in lost hours and increased financial hardship. Another potential explanation has been demonstrated by research examining health and economic threats. This research suggests that experiencing economic threats resulted in more individualistic and achievement-focused goals and less communal and public health-related goals. On the other hand, participants experiencing more health-related threats were more likely to engage in communal and public-health related goals [18]. This explanation is also consistent with the current findings related to fear of COVID-19, as those reporting more fear (health-related threats) were more likely to exhibit preventative behaviors and were also more likely to be vaccinated. COVID-19 related economic hardship was not related to preventative behaviors in Study 2 but there are several potential reasons for this. First, by August 2021, many of the economic uncertainties that were prevalent earlier in the pandemic had decreased. Additionally, the younger sample may have been more likely to still get financial support from parents, decreasing the salience of economic hardship, as many individuals in this sample may have not been responsible for supporting themselves or others financially.

Perhaps the most consistent findings in the current study related to normative beliefs, which had not been previously addressed in the COVID-19 literature. The extent that individuals believe that certain behaviors are normative and approved by their social group has been studied in prior public health research. For example, normative beliefs were predictive of smoking behaviors after accounting for a variety of other relevant variables [23]. Experimental research manipulating the extent that peers engaged in preventative behaviors found that exposing participant to information suggesting that their peers attend large gatherings and/or approved of attending large gatherings resulted in a decrease in participants’ avoidance of large crowds [26]. The current study found that in all analyses, participants normative beliefs were predictive of preventative behaviors, vaccine plans, and vaccination status. One potential reason these normative beliefs could be so predictive of behavior could relate to changes in interaction styles. The increased prevalence of communicating and obtaining information through social media outlets could result in normative beliefs becoming more distorted and more predictive of behavior. Recent research demonstrates that many of the most prevalent social media platforms consistently segregate their users and the information they are exposed to biased on their beliefs, resulting in an ‘echo chamber’ that could strengthen existing tendencies related to confirmation bias [40]. This tendency for individuals to have less exposure to contradicting ideas as a result of social media engagement could also lead to inaccurate views about how prevalent their own beliefs are related to public health and preventative behaviors. While being isolated from perspectives that differ from your own could be problematic in a variety of settings, that is especially the case regarding a global pandemic. Research has demonstrated that many individuals had opinions and beliefs related to COVID-19 that directly contradicted scientific findings [41]. This is potentially due to the large amount of COVID-19 disinformation, including conspiracy theories, that circulated largely in social media related to COVID-19 spread, prevention, and treatment [42]. The tendency for social media to serve as an echo chamber combined with the large number of fake news and conspiracy theories that were disseminated related to COVID-19 suggests that some individuals may have had greater exposure to misinformation than scientifically supported information.

Current findings related to pre-existing conditions were surprising but also consistent with previous research. Logically, having a pre-existing condition that places an individual at a higher risk for death or serious illness due to COVID-19 would result in increased preventative behaviors. In the current study, however, pre-existing conditions were not significantly related to preventative behaviors, vaccination plans, or vaccination status. This is consistent with previous research demonstrating that the presence of pre-existing conditions that place an individual at higher risk of serious illness was not consistently related to preventative health behaviors [28]. Porteny and colleagues [29] found a similar inconsistent finding, as only participants with obesity combined with other comorbid conditions were more likely to exhibit higher levels of preventative behaviors.

Findings related to personality demonstrated some consistency with prior studies but also potentially shed light on some aspects of personality and preventative health. Consistent with prior research [7,30,31], agreeableness and openness were both positively related to preventative behaviors. Conscientiousness was also positively related to peak preventative behaviors. Surprisingly, while conscientiousness and openness tended to be positively associated with preventative behaviors, vaccinated individuals scored significantly lower on both personality traits. This conflicting finding is unclear, and more research is needed to determine whether these personality traits are differentially associated with preventative behaviors and getting vaccinated or whether this finding was an anomaly, such as a type-1 error.

The personality trait that has the least consistent association with COVID-19 preventative behaviors seems to be extroversion. Some research has demonstrated findings suggesting that extraversion is positively related to following preventative behavior protocols aimed at preventing the spread of COVID-19 [30,32,33]. Other research has revealed a negative relationship between extraversion and preventative behaviors [30], while other studies report no significant relationship [34]. The current study could shed light on the inconsistent findings related to extraversion and COVID-19-related preventative behaviors. Extraversion was largely unrelated to preventative behaviors early in the pandemic among a non-student sample (Study 1). In Study 2, after vaccines were readily available and social distancing protocols were gradually lifted, the findings were interesting. Extraversion was not related to reported peak preventative behaviors. Current preventative behaviors, however, were negatively related to extraversion. This finding suggests that individuals high in extraversion may have been willing to exhibit preventative behaviors similar to others during the early stages of the pandemic. As vaccinations became available and restrictions were lifted, however, they were more eager to resume normal social functioning compared to their more introverted counterparts.

While the current study made several unique contributions to the existing literature, several limitations must be taken into consideration when interpreting the results. One of the biggest limitations involves the sample. The sample for Study 1 included participants across adulthood but was also very small, threatening generalizability. Still, several significant relationships were identified despite the lack of statistical power associated with such a small sample. While the Study 2 sample was considerably larger, it consisted of a primarily college-student sample lacking variability in age. It is important to interpret these findings in the context of the populations represented by these samples. Despite the limited size of the sample in Study 1 and the limited variability in age for Study 2, results across the two data collections were surprisingly consistent. The two studies revealed consistent findings related to interpersonal concern, fear of COVID-19, normative beliefs, pre-existing conditions, agreeableness, neuroticism, and openness. This level of consistency across different samples suggests the relationship between these variables and preventative behaviors may be stable across age and at different times during the pandemic.

Another limitation involves the measurement of peak preventative behaviors for Study 2. It is important to note that this was a cross-sectional study and the data related to peak and current preventative behaviors were self-reported during the Study 2 data collection. Reports of peak preventative behaviors during Study 2 are only as valid as the recollection and accuracy of reports made by participants.

Limitations aside, the current study made several unique contributions to the literature that could be important to future research and policy. Several variables important in understanding preventative behaviors and willingness to get vaccinated were identified. In Study 2, these variables accounted for over 50% of the variance in both peak and current preventative behaviors. The number of significant findings despite the small sample size of Study 1 also suggest strong relationships between these variables and preventative health. The finding that age (among the older samples) was significantly related to preventative behaviors but not to vaccination plans was surprising. Future research is needed to better understand why higher risk older adults might be more willing to engage in protective preventative behaviors but not more likely to plan on getting vaccinated. Findings of the current study combined with prior research related to economic hardship suggests that threats to economic stability can decrease the focus on public health while fear of health problems can increase efforts related to preventative behaviors. These findings suggest efforts to mitigate economic uncertainty associated with contagious pandemics combined with efforts to raise awareness of health risks could result in more widespread acceptance of public health protocols. Similarly, more efforts to identify which preexisting conditions are associated with higher risk could also result in more preventative behaviors from the most vulnerable members of the population. The strong relationship between normative beliefs with both preventative behaviors and vaccination status is especially concerning. The tendency for individuals, potentially through social media, to receive less exposure to contradicting viewpoints combined with confirmation bias is especially problematic. As a result, individuals with a tendency to resist public health efforts are likely to have a distorted view about the extent that their beliefs are normative and reinforced by others. An increased effort to expose individuals to conflicting information combined with more of an effort to equip individuals with the critical thinking ability to differentiate valid and invalid sources of information could potentially decrease this concerning trend.

## Figures and Tables

**Table 1:** Correlates of vaccine plans and preventative behaviors (Study 1; *N* = 44).

	Preventative Behaviors	Vaccine Plans
Vaccine plans	0.35 *	
Age	0.53 **	0.01
Interpersonal concern	0.34 *	0.13
COVID economic hardship	−0.40 *	−0.30
Fear of COVID	0.58 **	0.42 **
Normative beliefs	0.79 **	0.37 *
Pre-existing conditions	0.04	0.04
Extraversion	−0.01	−0.05
Agreeableness	0.19	0.44 **
Conscientiousness	0.31	−0.12
Neuroticism	−0.15	−0.01
Openness	0.51 **	0.16

Note. * *p* < 0.05; ** *p* < 0.01.

**Table 2:** Correlates of both peak and current preventative behaviors (Study 2; *N* = 274).

	Peak Preventative Behaviors	Current Preventative Behaviors
Age	−0.21 *	−0.08
Interpersonal concern	0.43 **	0.41 **
COVID economic hardship	0.05	0.07
Fear of COVID	0.29 **	0.41 **
Normative beliefs	0.58 **	0.72 **
Pre-existing conditions	0.02	0.11
Extraversion	0.03	−0.21 *
Agreeableness	0.25 **	0.24 **
Conscientiousness	0.17 *	0.10
Neuroticism	0.11	0.10
Openness	0.32 **	0.26 **
Vaccine Hesitancy	−0.27 **	−0.30 **

Note. * *p* < 0.05; ** *p* < 0.01.

**Table 3:** Regression analyses examining predictors of peak and current preventative behaviors (Study 2; *N* = 274).

Predictor	Peak Preventative Behavior		Current Preventative Behavior	
	*β*	*R^2^*	*β*	*R^2^*
Model		0.57 **		0.66 **
Age	−0.02		0.00	
Economic hardship	−0.05		0.02	
Pre-existing conditions	−0.05		−0.01	
Interpersonal concern	0.28 **		0.10	
Extraversion	0.05		−0.27 **	
Agreeableness	−0.07		−0.11	
Conscientiousness	0.30 **		0.22 *	
Neuroticism	0.12		−0.02	
Openness	0.27 **		0.24 **	
Fear of COVID-19	0.07		0.21 **	
Normative beliefs	0.40 **		0.64 **	
Vaccine hesitancy	−0.28 **		−0.31 **	

Note. * *p* < 0.05; ** *p* < 0.01.

**Table 4:** Summary of *t*-tests comparing participants based on vaccination status (Study 2; *N* = 274).

	Vaccinated		Unvaccinated		
	*M*	*SD*	*M*	*SD*	*t*
Age	19.78	6.03	20.37	6.75	−0.67
Economic hardship	2.48	1.30	2.38	1.23	0.60
Pre-existing conditions	2.49	1.22	2.33	0.78	1.09
Interpersonal concern	3.87	0.70	3.82	0.47	0.62
Extraversion	3.10	0.88	3.21	0.91	−0.95
Agreeableness	3.77	0.63	3.90	0.67	−1.62
Conscientiousness	3.74	0.61	4.01	0.54	−3.67 **
Neuroticism	3.10	0.79	3.02	0.76	0.81
Openness	3.36	0.67	3.57	0.62	−2.59 *
Fear of COVID-19	2.07	0.88	1.72	0.85	3.14 **
Normative beliefs	3.44	1.02	3.08	1.08	2.74 **
Vaccine hesitancy	2.22	0.71	3.08	0.77	−8.95 **
Peak Preventative Behaviors	4.49	0.90	4.02	1.10	3.69 **
Current Preventative Behaviors	3.49	1.15	3.03	1.22	3.02 **

Note. * *p* < 0.05; ** *p* < 0.01.

## Data Availability

All data related to the research reported in this article can be obtained by request from Kristopher J. Kimbler (kkimbler@fgcu.edu).
